# Diagnosis and management of 365 ureteric injuries following obstetric and gynecologic surgery in resource-limited settings

**DOI:** 10.1007/s00192-017-3483-4

**Published:** 2017-10-11

**Authors:** Thomas J. I. P. Raassen, Carrie J. Ngongo, Marietta M. Mahendeka

**Affiliations:** 1 P.O. Box 30125, 00100, Nairobi, Kenya; 2 P.O. Box 340, 00202, Nairobi, Kenya; 3 Mwanza, Tanzania

**Keywords:** Ureter, Ureteric, Injury, Iatrogenic, Fistula, Reimplantation

## Abstract

**Introduction:**

Ureteric injuries are among the most serious complications of pelvic surgery. The incidence in low-resource settings is not well documented.

**Methods:**

This retrospective review analyzes a cohort of 365 ureteric injuries with ureterovaginal fistulas in 353 women following obstetric and gynecologic operations in 11 countries in Africa and Asia, all low-resource settings. The patients with ureteric injury were stratified into three groups according to the initial surgery: (a) obstetric operations, (b) gynecologic operations, and (c) vesicovaginal fistula (VVF) repairs.

**Results:**

The 365 ureteric injuries in this series comprise 246 (67.4%) after obstetric procedures, 65 (17.8%) after gynecologic procedures, and 54 (14.8%) after repair of obstetric fistulas. Demographic characteristics show clear differences between women with iatrogenic injuries and women with obstetric fistulas. The study describes abdominal ureter reimplantation and other treatment procedures. Overall surgical results were good: 92.9% of women were cured (326/351), 5.4% were healed with some residual incontinence (19/351), and six failed (1.7%).

**Conclusions:**

Ureteric injuries after obstetric and gynecologic operations are not uncommon. Unlike in high-resource contexts, in low-resource settings obstetric procedures are most often associated with urogenital fistula. Despite resource limitations, diagnosis and treatment of ureteric injuries is possible, with good success rates. Training must emphasize optimal surgical techniques and different approaches to assisted vaginal delivery.

## Introduction

Ureteric injury is a potential complication of any pelvic operation [1]. Urinary tract injuries are among the most serious complications of gynecologic surgery [2] and can occur due to an inadvertent nick, cut, or tie of the distal ureter near the cervix as it crosses uterine vessels. Several authors have estimated the incidence of urinary tract injury, which includes ureteric injury, in well-resourced settings. One study found an incidence of 1 per 1000 gynecologic hysterectomies: 13.9 per 1000 after laparoscopic, 0.4 per 1000 after total abdominal, 0.3 per 1000 after subtotal abdominal, and 0.2 per 1000 after vaginal hysterectomy [2]. Other studies found incidences of 0.27 [3] and 0.9 [4] per 1000 cesarean sections (CS). Including all hysterectomies and routine gynecologic pelvic operations, the incidence is reported as 22 per 1000 [1]. These wide-ranging estimates may include radical hysterectomies and laparoscopic procedures, which involve a greater risk of damage to the ureters. Considering ureteric injuries as a group, two separate studies in the United States and Finland found that 50% and 75% of ureteric injuries follow gynecologic procedures, respectively [1, 2]. Ureteric injuries during hysterectomies more than doubled (0.29– 0.66%) in a comparison of two 5-year periods in England, attributable to adverse patterns of care [5].

The incidence of ureteric injuries following gynecologic and obstetric procedures in low-resource settings is not known. One study from Nigeria reported 3.6 per 1000 major gynecologic operations [6]. In such settings, obstetric operations are the main cause of ureteric injury [7–10]. The onset of symptoms and time of diagnosis of ureteric injury is often delayed following surgery [2]. Although the diagnosis can be made intraoperatively, injury is often identified after surgery with the help of excretory urography, retrograde pyelography, US (US), and cystoscopy. Ureteric injuries can be difficult to diagnose intraoperatively, particularly during emergency obstetric surgery when there is also concern about the baby [3]. Although ureteric injuries are reputedly the hardest of genitourinary surgical complications to diagnose [11], they can be identified during surgery, even in under-resourced settings [7, 12–14].

Most ureteric injuries are iatrogenic, being caused accidentally during CS or hysterectomy. A previous analysis compared iatrogenic ureteric injuries with other types of iatrogenic fistulas: vault and vesico-(utero)/−cervico-vaginal fistulas [15]. This analysis explores in greater depth a series of ureteric injuries and comprises diagnosis, treatment approaches, and results. It includes iatrogenic injuries previously considered as a separate group: ureteric injuries caused during vesicovaginal fistula (VVF) repair surgery [12, 13]. These postrepair injuries occur when providers inadvertently tie or cut a ureter when closing an obstetric fistula or are unable to incorporate the ureters in the fistula repair and thus leave them outside for later reimplantation.

This study analyzes the occurrence of ureteric injuries during pelvic surgeries in low-resource settings, examining patient demographics, surgical approaches, and results.

## Patients and methods

This retrospective study reviewed ureteric injuries repaired or attended to by the first or third author between June 1994 and December 2013. Surgeries took place in 11 countries: Tanzania, Uganda, Kenya, Rwanda, Malawi, South Sudan, Zambia, Ethiopia, Somalia, Bangladesh, and Afghanistan. Of the 6527 women who underwent fistula repair surgery, the authors encountered 353 with ureteric injury. All were included in this analysis. Data were collected verbally by the first and third authors on a standard form [16] before surgery and completed by the surgeon at the time of the surgery or shortly thereafter. Approval of this retrospective record review was granted by the Amref Health Africa Ethics and Scientific Review Committee.

Several approaches lead to the diagnosis of ureteric injury, with the most important being the patient’s history: the woman explains that after surgery she developed leakage, although she is still able to pass urine. In rare cases, the woman can recall left or right flank pain after surgery [4, 7, 12, 17]. In speculo examination is then performed with a dye test of 180 ml. If no dye is seen in the vagina, sometimes clear fluid is seen coming from the top of the vagina, suggesting ureteric injury. If no free fluid is seen, a three-swab test is done to confirm that leakage is most likely due to a ureteric injury.

If available, ultrasound (US) of the kidneys and ureters will likely reveal hydronephrosis and hydroureter on the affected side. An intravenous pyelogram (IVP) can help to visualize leakage, identify which side is affected, or show a nonfunctioning kidney. If a kidney does not appear in the IVP after 24 h, it has been damaged beyond repair and can either be removed (nephrectomy) [1, 7, 11] or the corresponding ureter tied. Some would recommend IVP as the first approach to diagnose which ureter is affected before beginning surgery [10, 11]. However, many underresourced facilities are not equipped to carry out an IVP or US; similarly, cystoscopy or retrograde urography, as recommended in high-resource settings, is unavailable [9, 11–13, 18, 19]. In such situations, the authors recommend that providers proceed with laparotomy after diagnosing a ureteric fistula and determine during surgery which ureter is affected.

Records of ureteric injuries were divided into three groups: (a) following an obstetric operation, i.e., CS, repair of ruptured uterus, or CS/hysterectomy; (b) following a gynecological operation, i.e., (sub)total abdominal hysterectomy; and (c) following a VVF repair. To be considered in the postrepair group, a woman must have been repaired by a surgeon who recognized a ureter injury during or immediately after the VVF repair surgery. Data analysis consisted of descriptive summaries of the sample. In Table 1, the two-sample* t* tests assume unequal variances, testing the null hypothesis that the difference between the demographic frequencies observed is zero between iatrogenic (obstetric and gynecologic) and postrepair groups. In Tables 2, 4, and 5, the reported probability is associated with the Pearson chi-square test.

**Table 1:** Demographic characteristics of ureteric injury patient groups

Variable	Iatrogenic – obstetric *n* = 242	Iatrogenic – gynecologic *n* = 62	Postrepair *n* = 49	t-test means comparison between iatrogenic and postrepair
Median age at time of repair	30.9 (*n* = 242)	43.1 (*n* = 62)	29.0 (*n* = 49)	*p* = 0.0050
Median duration of leaking (years)	2.5 (*n* = 237)	1.5 (*n* = 62)	6.6 (*n* = 49)	*p* = 0.0038
Median parity	4.5 (*n* = 241)	4.6 (*n* = 55)	2.4 (*n* = 49)	*p* = 0.0000
Median height (cm)	153.2 (*n* = 220)	158.2 (*n* = 52)	152.4 (*n* = 45)	*p* = 0.1704
Mean interval between procedure and leakage (days)	9.0 (*n* = 234)	10.8 (*n* = 50)	2.4 (*n* = 49)	*p* = 0.0000
Living with husband	75.1% (*n* = 241)	71.2% (*n* = 59)	56.2% (*n* = 48)	*p* = 0.0223
Attended at least some secondary school	11.5% (*n* = 235)	7.4% (*n* = 54)	2.0% (*n* = 49)	*p* = 0.0018

**Table 2:** Ureter affected

	latrogenic: obstetric	latrogenic: gynecologic	Postrepair	Total
Left	148 (61.2%)	35 (56.4%)	31 (63.3%)	214 (60.6%)
Right	90 (37.2%)	24 (38.7%)	13 (26.5%)	127 (36.0%)
Both	4 (1.6%)	3 (4.8%)	5 (10.2%)	12 (3.4%)
Total	242	62	49	353

Pearson chi-square test probability = 0.028

## Results

The 365 ureteric injuries comprised 246 (67.4%) following obstetric procedures, 65 (17.8%) following gynecological procedures, and 54 (14.8%) during surgical repair of an obstetric fistula. All except two anuric women presented with ureterovaginal fistulas: one after CS and one after total abdominal hysterectomy (TAH). In this series, no nephrostomy was done. 

Patient demographic characteristics indicated clear differences between obstetric, gynecological, and postrepair groups (Table 1). Women in the two obstetric groups were younger at the time of repair than women in the gynecologic group (*p* = 0.0044). Women whose ureteric injury occurred during obstetric fistula repair suffered longer before treatment (median duration of leaking 6.6 years; *p* = 0.0038). Women whose ureteric injuries arose during obstetric fistula repair had lower parity than women in the other two groups (*p* = 0.0000). Only four women in the postrepair group delivered at home, and eight babies in this group were born alive in the hospital. The interval between delivery and leakage was short in the postrepair group, with more than half of the women leaking immediately. The average interval to leaking was 2.4 days. Women with iatrogenic fistulas in the other two groups began leaking later after the causative procedure, with a median interval of 7 days and an average of 9–10.8 days.

Twelve women had bilateral ureteric injuries: four in the obstetric group, three in the gynecologic group, and five in the postrepair group. Among women whose injuries followed obstetric procedures, 156 had CS, 18 had ruptured uterus repairs, and 68 had CS/hysterectomy. Fifty-six (36%) of the 156 babies delivered by CS were stillborn. The sex of 292 babies delivered from the 290 women in the obstetric groups (242 CS, 48 postrepair groups) was as follows: 168 (57.5%) boys, 118 (40.4%) girls, and six (2.1%) in whom sex was not known. All 62 gynecological procedures were hysterectomies. Forty-four women in the iatrogenic obstetric and postrepair groups had concurrent obstetric fistula; both injuries were repaired at the same time. Thirteen women had another iatrogenic injury besides ureteric injuries: ten had vesico-cervico-vaginal fistulas (VCVF), and three had vault fistulas. Similarly, their injuries were operated simultaneously. Table 2 shows which ureter was affected in each group: the left was more often affected: 60.6% (214/353) vs 36.0% (127/353).

Abdominal reimplantation or repair of the ureter was the most common treatment approach (319/364), followed by abdominal tying of the ureter (29/364), vaginal reimplantation (13/364), and removing sutures around the ureter (3/364) (Table 3). Most women were managed by a ureteroneocystostomy (313/319), a simple end-to-side anastomosis of the ureter to the bladder mucosa with 4/0 Vicryl. A ureteric catheter was used as a stent, exeting through the bladder and abdominal incision. After several ureteric catheters came out during the operation, catheters were fixed inside the bladder to the mucosa with one 4/0 Vicryl stitch. The catheters were also fixed to the abdominal skin. No anitreflux procedure was used [10, 20]. This shortens the operation, which is important in low-resource settings and when operations are done under spinal anesthesia; results were good. In some women (29/353), the affected ureter was tied and severed after IVP showed no kidney function on the affected side. In a few cases (13/353), the ureter was reimplanted in the bladder via the vaginal route. In three cases a Boari flap was used and a psoas hitch in seven. Two women had an ureteroureterostomy (one left and one right) and another a right trans ureteroureterostomy. Two women with bilateral injuries were seen with anuria soon after their causative operations (CS and TAH) and sutures around three ureters were removed. When flow was seen into the bladder, ureters of one woman were not catheterized; in the other, one ureter was catheterized and the other ureter reimplanted.

**Table 3:** Kind of repair

Procedure	No.
Ureteroneocystostomy	313
Boari flap	3
Uretero-ureterostomy	2
Trans ureteroureterostomy	1
Vaginal ureter reimplantation	13
Tying of ureter (nonfunctioning kidney)	29
Untying of ureter	3
Failed laparotomy	1
Total	365

During abdominal reimplantation, several complications were encountered. Two women had injuries to the iliac vessels: one to the right iliac artery and vein, and one to the left iliac artery. All lesions were sutured with continuous 4/0 Vicryl. There were four lesions to small intestines and one to the rectum. These were all repaired, either by suturing (three cases) or resection and anastomosis (two cases). In two cases, the affected ureter was nicked then sutured with 4/0 Vicryl. We were unable to access the ureter in one very obese woman who was operated under spinal anesthesia. There was one immediate postoperative death due to asphyxia followed by cardiac arrest. The other postoperative complications were not serious: two women had a wound infection, one had pyelonephritis, one developed an incisional hernia, one had a blocked Foley catheter, one had urine retention, and two had unexplained fever and pain. Results were available for 351 women (Table 4) (one death and one instance of missing data). Overall, 326/351 (92.9%) women with ureteric injuries were completely cured, while 19/351 (5.4%) experienced some residual incontinence. In six women the operations failed, meaning the ureter could not be reimplanted or the obstetric fistula closed. Table 5 presents results by repair approach.

**Table 4:** Repair outcome

	latrogenic: obstetric	latrogenic: gynecologic	Postrepair	Total
Cured (closed and dry)	231 (95.8%)	60 (96.8%)	35 (72.9%)	326 (92.9%)
Healed (closed with remaining incontinence)	7 (2.9%)	2 (3.2%)	10 (20.8%)	19 (5.4%)
Not closed (failed)	3 (1.2%)	0 (0%)	3 (6.2%)	6 (1.7%)
Total	241	62	48	351

Pearson chi-square test probability = 0.000

**Table 5:** Outcome by repair approach

	Vaginal reimplantation	Abdominal reimplantation	Tying	Untying	Total
Cured (closed and dry)	8 (66.7%)	291 (94.2%)	24 (85.7%)	2 (100%)	325 (92.6%)
Healed (closed with remaining incontinence)	3 (25.0%)	15 (4.8%)	4 (14.3%)	0 (0%)	22 (6.3%)
Not closed (failed)	1 (8.3%)	3 (1.0%)	0 (0%)	0 (0%)	4 (1.1%)
Total	12	309	28	2	351

Pearson chi-square test probability = 0.008

Abdominal reimplantation was the most common approach and was successful in 94.8% of women.

## Discussion

Ureteric injuries are among the most serious complications of pelvic surgery. Our large sample, collected over nearly 20 years, demonstrates that diagnosis and treatment of ureteric injuries is possible in low-resource settings, with success rates greater than 90%. Unlike in high-resource contexts, obstetric procedures are most often associated with urogenital fistula. Demographic characteristics show clear differences between women with iatrogenic injuries and those with obstetric fistulas.

Overall surgical results were good: 92.9% were cured, while 5.4% were healed with some residual incontinence. Six surgeries failed (1.7%): three in the iatrogenic obstetric group and three in the postrepair group. Two women in the first group leaked from the first day after surgery (tying of left ureter and reimplantation of right ureter), but the cause of their leakage was not clear and they did not return for further treatment. The third woman failed for lack of access to the left ureter during laparotomy. In the postrepair group, two women had concurrent vesicovaginal fistulas that failed to close. The third woman was HIV-positive and started leaking from her abdominal incision.

Results are comparable with other series, although most series are smaller—ranging from four to 89 cases [1, 7, 8, 10–14, 17–19]. Some series mention only ureteric injuries after hysterectomy [1, 17–19], while others describe injuries after obstetric and gynecologic operations [7, 10–14]. Only three published studies describe series in which the majority of operations were not gynecologic hysterectomies [4, 7, 10]. In this series, most ureter injuries (246/353) were caused by obstetric operations. Women are now more likely to deliver in health institutions than in the past, especially when delivery is prolonged. A gynecologic hysterectomy is an elective procedure and is less commonly performed in remote district and mission hospitals.

In well-resourced settings, diagnosis is often made during surgery. If not, ureteric injuries are identified after surgery through excretory urography, retrograde pyelography, US, and cystoscopy. In the low-resource settings considered here, ureteric injuries were identified after obstetric or gynecological surgery. In the absence of an operation report for the initial surgery, it is impossible to say whether the surgeon knew about the ureter injury. In the postrepair group, ureteric injuries were generally diagnosed during surgery.

Several contributing factors complicate obstetric and gynecological surgery, including endometriosis, previous pelvic surgery (mainly CS), radiation, cervical myoma, and surgeon experience. Fourteen percent of women with obstetric ureteric injuries and 5% with gynecological ureteric injuries had a previous laparotomy, suggesting that surgeon experience is a very important contributing factor. Surgeons should not clamp indiscriminately when faced with adhesions or bleeding during surgery, as this can cause injury to the ureter where it runs very near to the cervix. Dissecting the bladder off the lower uterine segment should be sharp [21, 22]. Environmental realities such as poor lighting or a sudden blackout without backup electricity are not unusual in low-resource settings and may play a role. Another important contributing factor is that many women in these settings come to the hospital very late.

In the absence of more advanced technologies, the three-swab test is used to diagnose ureteric injuries. The intraperitoneal route was used, because in many cases the affected ureter was not known. After opening the bladder, both ureteric orifices were inspected. The one not producing urine was probed and found to be blocked, after which it was reimplanted. This pragmatic approach was successful in all cases. A simple end-to-side anastomosis was used for the ureteroneocystostomy. There is no need for an antireflux procedure in adults. As Hinman indicates in his* Atlas of Urologic Surgery*, the need for an antireflux procedure has not been demonstrated in adults requiring ureteral reimplantation, and a simple direct anastomosis may have fewer problems [10, 20]. Others have questioned the need for antireflux procedures in renal transplants, finding comparable results between approaches [23, 24]. Once the peritoneum is opened, the affected ureter is often seen as being thicker (more dilated) than the nonaffected ureter. This should be mentioned in the operation report. After incising the bladder, frusemide can be given IV to show which ureteric orifice is producing urine.

In this series, ureteric catheters (Ch. 5 or 6) were used as a stent, as they are less expensive than a double-J stent and can be removed 8–10 days after surgery, while the woman is still hospitalized. This contrasts with 6 weeks of double-J stent in environments where patient follow-up is more feasible. In 29 women, the ureter was ligated and severed because the kidney was not functioning on IVP. This practice has not been mentioned elsewhere in the literature. Twenty-six women were cured, while three had incontinence from the urethra. Nephrectomy in these women is not necessary (the same practice is followed in kidney transplantation). Almost one quarter (76; 24.3%) of the 313 ureteroneocystostomies were done by nonsurgeons, mostly gynecologists. The operation is not difficult if one observes surgical principles. When possible, a vaginal approach to ureteric repair is the least traumatic in our experience. It is feasible when the ureteric orifice is seen during vaginal examination and can be catheterized. However, results for the 13 women repaired using the vaginal route were suboptimal. Of the 12 women with available data, eight (66.7%) were cured. Three women (25.0%) healed, but they had concurrent fistulas with postrepair residual incontinence. One failure occurred when the concurrent fistula repair was not successful.

Some of the ten intraoperative complications were serious. Ureters can be highly adherent to iliac vessels due to inflammation and fibrosis. Dissection needs to be done very carefully. All three injuries were small nicks, which could be sutured once the bleeding was controlled with arterial clamps or finger pressure on either side of the lesion. The two ureteric lesions were also related to fibrosis around the ureters. Lesions to intestines were caused during dissection of severe adhesions between the intestines. These intraoperative complications are not found in the literature.

One woman from the obstetric reimplantation group died of asphyxia due to an anesthetic error: after extubation, the anesthetist did not check the woman, and her tongue blocked her airway. Two other studies each report one death: one due to renal failure after bilateral ureteroneocystostomy [7] and one due to pulmonary embolism [10]. Our other postoperative complications were minor. 

The interval between delivery and leakage for women in the postrepair group is short (mostly within 2 days) and statistically significantly different from the other two groups (*p* = 0.0000) [25]. This is because these women are leaking from their obstetric fistulas. Women in the other groups start leaking later, sometimes after 1 or 2 months. The idea is that it takes much longer for an injured ureter—cut, tied, or clamped—to start leaking. It takes time for sutures to be absorbed and urine to find its way through tissues to the cervix or vagina.

Several studies explored why the left ureter is more likely than the right to be injured during obstetric and gynecologic surgery [10, 11, 15] (Table 2). However, the different likelihoods of left vs. right ureteric injuries are not evident among women following a ruptured uterus repair (18) or CS/hysterectomy (68) (41 left, 44 right, 1 bilateral). A ruptured uterus presents a more complicated situation in which indiscriminate placing of clamps and sutures might be used. Interestingly, the greater risk to the left ureter is evident among women in the postrepair group, likely because most fistula surgeons are right handed, which makes vaginal dissection on the left more difficult than on the right.

Twelve women (3.4%) had bilateral injuries, a rate lower than other series, which report 5–13% [1, 7, 12, 17–19]. No explanation for the difference can be given, except that most series are much smaller than ours. Women in this series delivered more boys (168; 57.5%) than girls (118; 40.4%), similar to other reports [16, 25, 26]. There are two possible explanations: the higher rate of conception for males than females (106–7:100) and that male babies are on average 150 g heavier than female babies. Their head circumference may be a few millimeters larger than that of female babies, leading to prolonged, obstructed labor. Better parameters might be the weight and the size of the baby, but these are not known, especially if the baby is stillborn.

Of the 179 CS (including 17 from the postrepair group), 73 babies were stillborn. Other authors have urged the need to consider alternative delivery approaches for dead babies [10, 15], such as destructive delivery, to prevent accidental injury to the ureter during CS, as long as women continue to arrive late to the hospital with obstructed labor and a dead fetus. This is similarly an issue for women with obstructed labor who are not immediately attended to within health facilities. Even in cases where the baby is alive there are clear indications for alternative assisted vaginal delivery techniques, such as vacuum extraction and symphysiotomy [27, 28].

In the postrepair group, VVF repair attempts led to ureterovaginal fistulas. This might occur accidentally by injuring the ureters or if the ureters cannot be folded into the bladder during fistula repair. Two studies [12, 13] mention injury to the ureter after VVF repair. Demographic characteristics such as age, marital status, and height affirm that the postrepair population is similar to women with obstetric fistula [16, 29]. Only one woman in the postrepair group had an original fistula from gynecologic hysterectomy. Surgical results in the postrepair group were not as good as those in the two other groups. Although reimplantation might have been successful, women were still leaking from the urethra as a consequence of the VVF for which they underwent repair. This is postrepair incontinence, a serious problem after VVF repair. Characteristics of women in the postrepair group were similar to VVF patients described in the literature. For example, the median age of fistula development was 22 years [16, 30]. A lower education level is typical for those who suffer prolonged, obstructed labor. Like women in the obstetric group, they tend to be shorter than women in the gynecological group, although this is not statistically significant (*p* = 0.1704). Women who have had a surgery, such as CS or hysterectomy, are more likely to seek medical attention. Women in the postrepair group leak much longer than women in the other two groups, because they have already been operated once or more for their obstetric fistula.

Selzman describes differences between nonurological (gynecological and surgical) and urological injuries to the ureter [1]. Most injuries in his series were urological (42%). When injuries were detected during surgery, results of immediate treatment were better and fewer procedures were needed for both nonurological and urological injuries. However, the author did not differentiate between surgical and gynecological injuries. There were 56 (34%) gynecological injuries, all in the lower one third of the ureter, as in our series. Forty-seven cases were detected after surgery. In this series, all injuries were detected after surgery as uretero-(cervico)-vaginal fistulas, except in the two women with anuria and in some post-repair women. All women needed just one procedure to correct the injury. This suggests that obstetric/gynecologic injuries are less serious than urological injuries, even if dealt with years after the causative surgery.

Selzman reported a decreased incidence of ureteric injuries during obstetric and gynecologic surgeries due to improvement of surgical technique and increased surgeon awareness about the risk [1]. However, Hilton mentions that the incidence of ureteric injuries after hysterectomy has doubled after comparing two 5-year periods [5]. The frequency of ureteric injuries seems to be increasing in low-resource settings, because more CS are being done than before, and most are done by nongynecologists.

There are limitations to this study. The information on the records comes from the women, and although they usually know their obstetric and gynecological history well, in most cases many years passed before they received treatment. Although an increase in body mass index is associated with an increase in CS rate, we do not have information about women’s BMI at the time of their deliveries. There has been hardly any follow-up. In a few cases, women returned after 3 months, but only for clinical examination, without postoperative US or IVP. Limited conclusions can be drawn about the incidence or prevalence of ureteric injuries following obstetric and gynecological operations, since this series is based solely on the experiences of the authors.

Ureteric injuries during obstetric and gynecological operations are probably increasing. Training for specialists, medical officers, and paramedical staff is required in proper techniques for performing these operations, like using sharp dissection and locating and, if necessary, dissecting the ureters. Early detection and repair is better than late repair. There are clear indications for alternative ways to deliver babies, depending on whether the baby is alive or dead. Vacuum extraction, symphysiotomy, and destructive delivery techniques should remain in the training curriculum as long as women continue to arrive with prolonged obstructed labor in resource-limited settings. The experience of the attending doctor is the most important factor in minimizing ureteric injuries.
